# Hepatitis A Virus and Hepatitis E Virus as Food- and Waterborne Pathogens—Transmission Routes and Methods for Detection in Food

**DOI:** 10.3390/v15081725

**Published:** 2023-08-12

**Authors:** Katalin Nemes, Sofia Persson, Magnus Simonsson

**Affiliations:** European Union Reference Laboratory for Foodborne Viruses, Swedish Food Agency, Dag Hammarskjölds väg 56 A, 75237 Uppsala, Sweden; sofia.persson@slv.se (S.P.); magnus.simonsson@slv.se (M.S.)

**Keywords:** hepatitis A virus, hepatitis E virus, foodborne transmission, viral detection, RNA, detection in food, foodborne outbreak, foodborne disease, bivalve molluscan shellfish (BMS)

## Abstract

Foodborne viruses are an important threat to food safety and public health. Globally, there are approximately 5 million cases of acute viral hepatitis due to hepatitis A virus (HAV) and hepatitis E virus (HEV) every year. HAV is responsible for numerous food-related viral outbreaks worldwide, while HEV is an emerging pathogen with a global health burden. The reported HEV cases in Europe have increased tenfold in the last 20 years due to its zoonotic transmission through the consumption of infected meat or meat products. HEV is considered the most common cause of acute viral hepatitis worldwide currently. This review focuses on the latest findings on the foodborne transmission routes of HAV and HEV and the methods for their detection in different food matrices.

## 1. Introduction

The estimated number of cases caused by foodborne diseases is 600 million each year, leading to 420,000 premature deaths worldwide [1]. Food can be unsafe for human consumption due to the presence of pathogens such as bacteria, viruses besides parasites and chemical or physical substances [2]. Human pathogenic viruses represent a frequent causative agent of foodborne diseases, having a significant impact on human health and the economy. Although numerous viruses may be foodborne transmitted, noroviruses and hepatitis A virus (HAV) are the major causative agents of foodborne viral outbreaks. Hepatitis E virus (HEV) is a significant cause of acute viral hepatitis worldwide. Additionally, it is an emerging pathogen in economically developed countries, and the foodborne transmission of this zoonotic virus appears to be the major route in Europe (HEV-3) [3,4]. Both HAV and HEV are small RNA viruses but differ in genomic organization and structure. 

Hepatitis A virus is a positive-stranded RNA virus belonging to the genus *Hepatovirus* of the *Picornaviridae* family [5,6]. HAV is a small virus with a 27–32 nm diameter virion. Two different forms of this infectious virus can be found: the naked, non-enveloped HAV virions which are excreted in the stool, and the quasi-enveloped virions (eHAV), which are released non-lytically from infected cells. eHAV are found in the blood of infected patients or in the supernatant of infected cell cultures [7,8]. HAV is a causative agent of acute infection of the liver; the clinical course of infection ranges from mild to severe while children (<6 years) are often asymptomatic. Symptoms can include fever, malaise, loss of appetite, diarrhoea, nausea, abdominal discomfort, dark-coloured urine and jaundice (a yellowing of the eyes and skin) [2]. Infection by HAV leads to lifelong immunity and does not result in chronic infection or chronic liver disease [9]. The HAV infection is rarely fatal; the WHO estimated that in 2016, 7134 people died from HAV worldwide (accounting for 0.5% of the mortality due to viral hepatitis [1]). 

HAV is classified into six genotypes; three genotypes infect humans (I, II and III) and IV, V and VI infect simians. Genotypes I, II and III are divided into seven subtypes (IA, IB, IC, IIA, IIB, IIIA and IIIB). Worldwide, genotype IA is the most reported, which is followed by III, while genotype II has been rarely reported. For the latter, genomic information is sparse [10,11]. 

Hepatitis E virus (HEV) is a small single-stranded RNA virus that belongs to the *Hepeviridae* family in the *Orthohepevirus* genus [12]. Similar to HAV, two different HEV particles exist: the non-enveloped particles that are mainly found in faeces and the quasi-enveloped particles that can be found in serum and cell culture supernatant [13]. Non-enveloped particles showed higher infectivity in cell culture and in uPA-SCID mice model compared to enveloped particles [14,15].

HEV is classified into eight genotypes; HEV-1-4 and HEV-7 have been shown to infect humans; HEV-5 and 6 have been shown to infect only wild boar. HEV-3 infects domestic pig and wild boar, while HEV-7 and HEV-8 has been proposed as a zoonotic camel variant [16]. HEV-3 and HEV-4 are considered zoonotic [17,18]. HEV genotypes are further divided in subtypes [19,20].

HEV-1 strains are endemic to Asia and most countries in sub-Saharan Africa, where it is the main cause of recurrent epidemics. Moreover, HEV-1 is causing large outbreaks in India [21]. HEV-2 is present in Mexico, Nigeria, Chad, Sudan, and the Central African Republic [22]. HEV-3 has a worldwide distribution (Europe, the United States, and other North American countries, Central and Southern Japan, New Zealand, and Australia) while HEV-4 can be found predominantly in Asia (China, northern Japan, and India) [23]. HEV-5 and HEV-6 were detected in Japan [24]. So far, HEV-7 has been identified in the United Arab Emirates, Somalia, Sudan, Egypt, Kenya, Pakistan, Israel, Saudi Arabia and Djibouti [16]. HEV-8 was detected in China [25].

Most of the HEV infections are asymptomatic but HEV can cause symptomatic infection leading to a self-limited, acute hepatitis [26]. HEV can become chronic in immunosuppressed people [27,28]. HEV-1 and HEV-2 can cause severe disease and fulminant hepatitis; pregnant women are affected primarily [29]. HEV infection in pregnant women shows rapid virus replication and has a high incidence of developing fulminant hepatic failure (FHF) or acute liver failure (ALF) [30]. The mortality rate in pregnant women is often 30% or higher particularly in the third trimester [31]. Wang and colleagues identified two mutations in the ORF1 of HEV-1 that enhanced viral replication and may be associated with FHF [32]. Rarely, HEV can lead to ALF not only in pregnant women but also in elderly patients, patients with leukaemia or coinfection with other hepatotropic viruses [33,34,35]. HEV-3 and HEV-4, on the other hand, do not appear to cause fatal infections with fulminant hepatitis in pregnant women, but this has to be confirmed in future studies [36]. However, HEV-4 was shown to be associated with adverse pregnancy outcomes in pregnant women [37,38]. Rarely, acute and chronic and otherwise symptomatic and asymptomatic HEV-3 infections can cause neurological disorders (e.g., neuralgic amyotrophy and Guillain–Barré syndrome) [39].

## 2. Transmission Routes

The shedding of foodborne viruses originates from the human gastrointestinal tract, and their presence in water and food is a result of contamination by sewage, poor hygiene, or contamination by food handlers [40]. Food can be contaminated at any stage from farm to table; however, often, foodborne viral infections can be traced back to infected food handlers [41]. Foodborne viruses (including HAV and HEV) are typically highly resistant to environmental factors, such as low pH (acidity) and heat [42,43]. They can remain infectious for over a month in food and water [44,45].

### 2.1. Foodborne Transmission of HAV

The main route of HAV transmission is from person-to-person by the faecal–oral route. HAV outbreaks occur from time to time in high-risk populations, such as men who have sex with men (MSM, sexual transmission) and persons who use injection drugs (bloodborne transmission) [46,47]. Vertical transmission of HAV (from the mother to the foetus) is uncommon, although there are few case reports published [48,49]. In addition, HAV can be transmitted by the ingestion of contaminated food or water. Closed environments and restaurant caterings results in the most common setting of HAV outbreaks. Most European countries are considered as non-endemic or have low endemicity [50], so infection is often related to recent travel to countries with higher rates of HAV. In addition to travelling, HAV infections and outbreaks are frequently associated with imported food products (Table 1).

Berries, green-leaf salads and bivalve molluscan shellfish (BMS)—that are often eaten unprocessed—are the most common food matrices implicated in HAV outbreaks. Ready-to-eat meals contaminated by a food handler or using a contaminated ingredient (e.g., berries on cakes) can cause outbreaks as well. A harmonised investigation in three European countries demonstrated the presence of human enteric viruses in the leafy green vegetable supply chain; however, HAV was only detected in samples from the primary production phase [51]. Due to the complexity of food supply chains and the long incubation time of HAV (with a mean of about 30 days) [52], it is often difficult to identify the actual contaminated source. Table 1 summarises the lately published outbreak studies of HAV containing the investigations in which the implicated food or food handler was confirmed by PCR. In addition to dates and clams, the outbreaks were related to frozen berries (often imported), lettuce and dried tomatoes (Table 1).

**Table 1:** HAV outbreaks associated to food published in the last 15 years.

Year	Genotype	Implicated Food	Imported ^1^	No. of Cases	Outbreak Location	Reference
2021	IB	medjool dates	Y	31	England and Wales	[53]
2021	IB	medjool dates	Y	6	Australia	[54]
2019	IA	jogaejeot (fermented clams)	N		South Korea	[55]
2019	IA	blackberries	Y	20	USA	[56]
2018–2022	IB	strawberries	Y	65	Germany	[57]
2018	IB	strawberries	Y	20	Sweden and Austria	[58]
2018	IB	frozen pomegranate	Y	30	Australia	[59]
2017	IB	frozen raspberry/blueberry	Y	14	The Netherlands	[60]
2016	IA	raw scallop	Y	292	Hawaii	[61]
2016	IA	frozen strawberries and blackberries	Y	7	New Zealand	[62]
2015	IA	mixed frozen berries packed	Y	67	Australia	[63]
2014	IA	kava drink	N	4	Australia	[64]
2013–2014	IA	mixed frozen berries	Y	1803	Italy, Germany, Ireland, Norway, Austria, Poland, Netherlands, Bulgaria, Denmark	[65,66,67]
2013–2014	IA	frozen berry mix cake	Y	33	Norway	[68]
2012–2013	IB	frozen strawberries	Y	103	Denmark, Finland, Norway and Sweden	[69]
2012	IB	pomegranate seeds, a component of frozen fruit blend	N	9	Canada	[70]
2012	IA	mussels	N	9	The Netherlands	[71]
2011	IB	semi-dried tomatoes	nd	8	The Netherlands	[72]
2011	IB	semi-dried tomatoes	nd	7	England	[73]
2010	IB	semi-dried tomatoes		13	The Netherlands	[74]
2010	IB	semi-dried tomatoes	Y	59	France	[75]
2009	IB	semi-dried tomatoes	Y and N	562	Australia	[76]
2008	nd	lettuce	nd	22	California, USA	[77]
2008	IB	Raw clams (Coquina clams)	Y	100	Spain	[78]
2008	IIIA	lettuce or carrot	N	nd	South Korea	[79]

^1^ Y (yes)/N (no)/nd (not defined).

Bivalves are filter feeders and can therefore bio-accumulate and concentrate microorganisms and viruses from water. Hepatitis A outbreaks are rarely reported in association with the consumption of contaminated bivalve shellfish [80,81], which is possibly due to the low and decreasing incidence of HAV in the European population [82] and due to the long incubation time, making trace-back difficult. The presence of HAV in BMS varies over time and place. Dirks and colleagues studied the presence of HAV (besides norovirus) in bivalve molluscs in the Netherlands between 2013 and 2017. Among the total of 228 oyster and 392 mussel samples, the presence of HAV RNA was detected in only one mussel sample (0.3%, genotype IA) [83]. In the Campania region (Southwest Italy), 289 samples from shellfish production areas and other locations were tested between 2015 and 2017; 8.9% of the samples tested positive for HAV [84]. Shellfish samples (108, fresh and frozen) were collected from three harvesting areas and from restaurants, fish markets, and shellfish markets in Sicily; 13% of the samples tested positive for HAV (14 HAV-positive samples, one HAV 1A and 13 HAV 1B) [85].

In a study assessing the trend of HAV, after a contamination in BMS caused by a community outbreak, a minimum of 5 weeks was required to reduce viral loads to undetectable levels. Moreover, according to their regression analysis, 2–3 months may be required to ensure the removal of residual viral particles present in concentrations below the detection limits of the analytical method [86]. 

Rarely, drinking water can be a source of HAV infection; several HAV outbreaks have been documented and linked to faecally polluted drinking or recreational water [87].

### 2.2. Transmission of HEV

Unlike HAV, human-to-human transmission of HEV is uncommon. Secondary attack rates among household contacts of HEV cases was shown to be very low (0.7–2.2%), while it was 50–75% in the case of HAV infections, which is another enterically transmitted virus [88]. The most common sources of HEV infections that occur in humans are contaminated water or food. However, the number of reported transfusion-transmitted HEV (TT-HEV) cases, in blood donation recipients, is also on the rise [89]. In addition, the maternal–foetal transmission of hepatitis E virus has also been reported [90,91].

#### 2.2.1. Waterborne Transmission

HEV-1 and HEV-2 are human specific, and large HEV outbreaks occur due to contaminated water consumption in low-income countries [92,93,94]. An investigation of a tap water-mediated HEV outbreak in China suggests that sporadic cases of waterborne outbreaks of HEV-4 may occur in industrialised countries [95]. Sewage of human and swine origin can be a potential transmission route for HEV, and few infected people can contaminate municipal wastewater treatment plants (WWTP), increasing the potential for further spread of the virus [96]. Coastal waters can become contaminated by human sewage and manure runoff, and therefore, HEV can accumulate in shellfish produced close to land [97,98,99]. The detection of HEV-3 in surface and drinking water indicates the potential risk of HEV-3 transmission through drinking water [100,101,102].

#### 2.2.2. Zoonotic Transmission of HEV

Meat consumption as a source of HEV infection of humans was first described in 2005 [103]. Foodborne transmission in Europe is linked to the consumption of HEV-contaminated pork meat products (liver sausages, salami), undercooked wild boar meat and raw venison. Zoonotic transmission of HEV-3 (predominant in Europe and in America) and HEV-4 occurs frequently, and pigs represent the main reservoir for zoonotic HEV [104]. 

The numerous contact points between humans and domestic and wild animals from breeding to the food industry redound HEV transmission. The prevalence of HEV in pigs varies between pig farms, farming systems and countries. Generally, both the between-herd and within-herd prevalence of HEV is high (between 20 and 75%), indicating the frequent exposure of pigs to the virus [105,106,107,108,109]. Boxman and colleagues found a high prevalence (16%) of acute HEV infection in pigs in Dutch slaughterhouses [110]. The length of the fattening period has been shown to affect the risk of HEV; a longer fattening period may lower the risk of HEV in shedder animals at slaughter and thus reduce the risk of food contamination [111]. 

The seroprevalence of immunoglobulin G (IgG) against HEV (anti-HEV IgG) ranges between 10 and 20% in the European human population [112,113,114,115], and exposure to pigs is associated with a significantly higher seroprevalence (in people with contact to pigs) compared with non-exposed humans [116]. A high prevalence of anti-HEV antibodies among swine slaughterhouse workers and farmers has been reported, suggesting an occupational risk of HEV infection [117,118,119,120]. Wu and colleagues studied the risks of HEV infection in workers along the meat supply chain [121]. Their analysis showed that the human HEV infection risk increased along the pork supply chain, with the highest risk at pig slaughterhouses and pork markets; in contrast, no significant higher risk was observed among poultry workers. 

Outbreaks of HEV-3 are primarily associated with the consumption of pork products, especially undercooked pork liver or sausages [122,123]. Pig liver is more frequently positive for HEV, while muscles are less often contaminated by HEV-RNA [107]. In accordance with that, the presence of HEV is the highest in products containing liver, which is followed by raw meat sausages (Table 2). Table 2 summarises the recently published studies of the presence of HEV RNA in commercially available pork products and meat cuts. A parametric stochastic model estimating the risk of foodborne exposure showed that products containing liver pose the highest risk at the individual level [124].

Studies on the efficiency of inactivation methods for HEV during meat processing are quite limited [139]. According to a study by Johne and colleagues, high hydrostatic pressure processing (HPP) can be considered as a treatment method for decreasing the risk of foodborne HEV transmission [140].

In addition to swine, wild boar is also an important natural reservoir for the zoonotic transmission of HEV around the world. Since HEV infections are mostly asymptomatic, only two outbreaks associated with the consumption of undercooked venison and wild boar has been published so far [103,141]. Table 3 summarises an updated list of survey studies published of the presence of HEV in wild boar. The prevalence of the virus varies greatly between different geographical regions, as does as the number of samples tested in these surveillance studies.

Although swine and wild boar are the main reservoirs of HEV-3-6, several other animal species act as an HEV host, including domestic and wild ruminants. The first identification of HEV RNA and evidence of zoonotic transmission of HEV in deer has been published in 2003 [142]. Since then, numerous surveillance studies were published on molecular and serologic screening of serum and faecal samples from domestic and wild ruminant species. The virus is present in deer (red deer, *Cervus elaphus*; roe deer, *Capreolus capreolus*; fallow deer, *Dama dama*) and in chamois (*Rupicapra rupicapra*); however, the prevalence of HEV varies in different geographic regions [143,144,145,146,147]. A high seroprevalence of HEV has been observed in wild reindeer (23.1%) and moose (30% and 19.5%); furthermore, HEV exposure in muskoxen has been reported [148,149]. In a study from Finland, HEV seroprevalence was 9.1% (31/342) in moose and 1.4% (1/70) in white-tailed deer, but HEV RNA could not be detected from samples of seropositive animals [150]. HEV RNA was detected in 7.69% of the moose samples (n = 13) in a surveillance study in Lithuania [147].

HEV-3 and HEV-4 have been detected in bovine liver as well, suggesting that bovine livers may be involved in the zoonotic transmission of HEV to humans [151,152]. Moreover, HEV-3 is also present in sheep and goat populations in Italy [143,153]. Recently, sporadic infections with rabbit HEV (raHEV) have been reported, and immunosuppressed patients (solid organ transplant recipients) have been infected with rabbit HEV (genotype 3ra) [154,155]. In France, 5 of 919 (0.5%) HEV-infected patients during 2015–2016 were infected with a rabbit HEV strain. The source of infection was unclear because none of the patients had direct contact with rabbits. The HEV-3ra infections could be the result of foodborne or waterborne infections [156].

Predominantly, the HEV variants causing human infections belong to HEV species A (*Orthohepevirus* A, HEV-A) [12]. *Orthohepevirus* genotype 1 (HEV-C1) circulates in rats and was first isolated in Germany in 2010 [157]. HEV-C1 was earlier considered unable to infect humans, but since then, several rat hepatitis E virus (HEV) cases have been identified in humans [158,159,160,161,162]. Despite the occurrence of HEV-C1 infections in humans, several studies indicating that HEV-C1 infections are very rare among several populations in Europe [163,164,165]. Furthermore, the transmission route of HEV-C1 between rats and humans is elusive. Rat meat consumption is uncommon, suggesting other infection sources and transmission patterns (e.g., rat infestation in domestic premises, water contamination). Further epidemiological investigations would help to identify infection sources and transmission patterns.

HEV-3 and HEV-4 have been found to be excreted in the milk of ruminants [166,167,168]; however, data on the potential milk-borne transmission are still lacking or/and conflicting. The presence of HEV in milk samples from dairy farms was investigated in Germany, but HEV-specific RNA could not be detected in the 400 bulk milk samples [169]. The different results can possibly be due to the differences in the genotype distribution and the differences in the farming systems. Thus, further investigations on the prevalence of HEV in milk could facilitate the understanding of its risk of zoonotic transmission. The zoonotic infection by HEV-7 in a human liver transplant patient who had consumed camel milk demonstrates the ability of this virus to infect humans.

**Table 3:** HEV RNA detection in wild boar.

Year of Sampling	Species	Country	No. of Samples	HEV RNA Prevalence; Genotype and Subtype	Reference
2018–2022	wild boar	Portugal	144 stool samples	2.80%; HEV-3e, m	[170]
2017–2022	wild boar	China	599, faecal or serum samples	2.2%; HEV-4a, d, h	[171]
2019–2020	wild boar	Italy	86 liver	26.70%; HEV-3c	[172]
2019–2020	wild boar	Italy	156 livers	5.12%; HEV-3c	[173]
2013–2017	wild boar and deer	Germany	1961 wild boar, 559 roe deer, 736 red deer, 316 fallow deer	wild boar 13.3% and deer from 4.2%; HEV-3c, f, i	[174]
2012–2021	wild animals, including 15 species	Japan	3489 serum samples	1.2% of wild boar, 0.06% of Sika deer, HEV-3a, b, k HEV-4j	[175]
1997–2020	wild sika deer (Cervus nippon)	Japan	395 serum and 199 liver samples of 405 sika deer	0.20%; HEV-3b	[176]
2019	wild boar	Germany	liver, faeces, and muscle samples	mean 8.14%; HEV-3a, c, e, f, k, i	[177]
2016–2020	wild boar	Italy	611 livers and 88 paired lungs	2.45% of livers, 1.13% of lungs; HEV-3n	[178]
2015–2020	wild boars, red deer, roe deer and chamois	Italy	602 wild boar liver, 228 ruminant liver	6.97%; HEV-3a, f	[179]
2015–2019	wild boar	Poland	470 liver, 433 faeces	12.1% of liver, 6.2% of faecal samples	[180]
2018–2019	wild boar	Bulgaria	32	12.50%	[181]
2013–2019	wild boar	Japan	1803 serum samples, 1519 liver tissues and 42 gallbladder	3.90%; HEV-5	[182]
2015–2016	wild boar	Korea	1859 wild boar bloods	1.29%; HEV-3a, HEV-4a, d	[183]
2010–2017	swine and wild boars	Croatia	720 tissue and/or blood samples	11.50%; HEV-3a, e	[184]
2016–2017	wild boar	Italy	92 livers	52.20%; HEV-3c, f	[185]
2013–2015	wild boar	Romania	45 liver, 5 spleen	18%; HEV-3	[186]
2011–2012	wild boar	Portugal	80 liver samples, 40 stools	25% of livers, 10% of stools; HEV-3e	[187]
2003–2010	wild boar, Iberian pig, deer	Spain	287	10.12% of wild boar, 16.05% of red deer, 0% of Iberian pigs	[188]
nd	wild boar	Italy	594 serum and 320 liver	4.9% of liver, 0% of serum samples; HEV-3e, f	[189]
nd	wild boar	Sweden	159 blood samples	8.17%; HEV-3f	[190]

Genotypes 7 and 8 should be considered as potential human pathogens [191]. A review has been recently published, summarising the current scientific knowledge in HEV detected in milk [192].

## 3. Methods Used for Detection of HAV and HEV in Different Food Matrices

The low virus concentration and the complexity of food matrices make it difficult to detect viruses in food samples. In general, the virus detection procedure contains three main steps: (1) virus extraction from food matrices, (2) nucleic acid extraction and (3) detection by PCR-based methods. 

The first step of the virus detection is virus extraction, which includes the separation and concentration of viruses from food matrices. Methods for the extraction depend on the food composition; three main categories can be distinguished [193]. The first category is composed of carbohydrate- and water-based foods (fruits and vegetables). The second category includes fat- and protein-based foods (ready-to-eat products). Shellfish belongs to the third and separate food category due to the accumulation and concentration of viral particles and other pathogens in the shellfish digestive system. Stals and colleagues have summarised the variety of protocols for virus extraction from food samples [194]. 

The RNA extraction is mostly performed with commercially available kits that are based on the Boom nucleic acid extraction method using the disruption of viral capsids with guanidine thiocyanate and adsorption of viral RNA to silica magnetic beads or columns with silica [195]. Earlier, RNA was extracted by phenol–chloroform, which ischeaper, but produces hazardous by-products. RT-qPCR is widely used for virus detection because of its sensitivity and specificity. In addition, it delivers quantitative data if standard curves or reference values are used. On the other hand, the PCR is an enzymatic reaction and therefore is sensitive to inhibitors. Food samples are complex and often contain PCR inhibitors. Therefore, proper controls are needed to avoid false negative results (due to inhibition) and false positive results (due to cross-over contamination). Inhibition can be monitored using an external control (EC) RNA, as described, for example, in ISO 15216-1:2017 [196]. The degree of inhibition is measured by comparing the Cq value of a sample well spiked with EC RNA with the Cq value of a control well containing an equal amount of EC RNA in nuclease-free water. Typical PCR inhibitors in food matrices are phenols, polysaccharides (berries), pectin, polyphenol, xylan (green leafy vegetables), algae, and glycogen (bivalves) (summarised by [197]). 

### 3.1. Methods for HAV Detection

A standardised method is available for norovirus and HAV detection in food and has been validated in seven food matrices: bottled water, food surfaces, oysters (*Magallana gigas*, earlier: *Crassostrea gigas*), mussels (*Mytilus edulis*), raspberries, lettuce and green onions [196]. In addition to the validated, high-risk food categories, the standardisation and validation of detecting viruses from other food matrices is needed. For multicomponent foodstuff samples, methods have been described that can be used to detect viruses in composite food products for routine diagnosis [198,199,200]. 

### 3.2. Methods for HEV Detection

Unlike HAV and norovirus, no standardised method has been described so far for the detection of HEV in food samples; however, the standardisation process for the detection of HEV has recently been initiated by the International Organisation for Standardisation (ISO; ISO/TC 34/SC 9/WG31 Hepatitis E virus). For pork products, several RT-PCR methods have been described to detect HEV; a recently published review discusses these methods, focusing on the successful use in subsequent studies and surveys [201]. A validated method for HEV RNA detection in meat and meat products has been described by Althof and colleagues [202]. The method was validated in a ring trial with nine independent laboratories using artificially contaminated pork liver sausage samples. The method is currently the basis for the development of a new ISO standard in ISO/TC 34/SC 9 WG 31 (Magnus Simonsson, personal communication). Contaminated milk is a potential risk source for HEV infection, so the assessment of HEV in the milk is of great importance for consumers’ safety [166,203]. A study has been published recently on the detection of HEV in milk-based food products, showing that the HEV molecular assay could be improved by the selection of the extraction procedure that matches with the milk matrix. In addition, the removal of inhibitory substances such as fat and casein from the milk sample increased the performance [204].

### 3.3. Digital PCR Vs. qPCR

In recent years, digital PCR (dPCR) has been recognised as a useful alternative to qPCR for detecting and quantifying viruses in food samples [205,206,207]. The idea of digital PCR was described already in the 1990s (then referred to as limited dilution PCR [208]), but it was not until the 2010s that the technique became widely commercially available. The sensitivity of the reverse transcription digital PCR (RT-dPCR) assay was similar to that of RT-qPCR, and the methods agreed well in quantification of the presence of HEV in naturally contaminated pig products [209]. Similar to qPCR, dPCR allows the simultaneous quantification of different targets. HAV and norovirus genogroup I and II were quantified simultaneously by triplex droplet reverse transcription digital PCR in food, drinking water, and faecal samples [210]. In general, RT-dPCR can be more expensive and time consuming compared to RT-qPCR, but no external calibration curves are needed for the quantification. In addition, compared to RT-qPCR, RT-dPCR generally offers higher precision in quantification [211] and is less affected by inhibitors [212,213] and primer–template mismatches under certain conditions [214,215,216].

### 3.4. Molecular Typing

The typing of viral strains allows the identification of the possible source of foodborne outbreaks [217]. Moreover, it helps with studying the epidemiology and transmission routes of the viruses. Online genotyping tools have been developed to identify the HAV and HEV genotypes of a nucleotide sequence (https://www.rivm.nl/mpf/typingtool/hav/ (accessed on 24 April 2023); https://www.rivm.nl/en/Topics/H/HEVNet (accessed on 24 April 2023)). 

Next-generation sequencing (NGS) is a sensitive and widely used technique for the molecular typing of pathogens. Yang and colleges identified inter- and intra-host variants of HAV in the clinical specimens and under laboratory culture conditions using NGS [218]. Lately, a multiplex PCR-based NGS was implemented to define HAV genotypes from hepatitis A patients [219]. NGS is becoming frequently applied in food sample testing, primarily for norovirus typing [220,221]. Only few studies have been published so far on the application of NGS for the typing of HAV and HEV in food matrices. The majority of the NGS methods for foodborne virus analysis are amplicon-based and incorporate an RT-PCR amplification step (amplification of subgenomic regions, such as the VP1/P2B junction in case of HAV). These methods can be used for the identification of transmissions among cases with known epidemiological association. As an example, HAV was detected and identified as a source of outbreak from frozen berries by NGS in northern Italy [222]. Yang and colleges developed a sensitive and amplification-independent method for virus characterisation. Celery samples were used as a food matrix, and they were inoculated with high and low copies of viruses (norovirus and HAV) as a single or as a multi-strain mixture [223]. Compared to amplicon-based sequencing, the whole-genome sequencing methods offer a more accurate tracking of virus strains. In a study by Vaughan et al., HAV genetic identity was precisely determined using whole genome (WG) sequences using samples from food-borne outbreaks besides non-outbreak-related samples [224].

Both the ORF 1 and ORF2 regions are used for molecular typing of HEV for Sanger sequencing and NGS, and different subtypes can be determined [130,225,226,227]. HEV-3 subtypes c, e and f have been shown to be the most prevalent subtypes in Europe [4]. Investigations on the HEV strains circulating in pig farms showed that each farm has its unique HEV-3 strain, and the same strain is present in the farms for several years [109,190]. This uniqueness can be helpful in outbreak investigations by facilitating the source tracking.

Sanger sequencing and NGS are still the primarily used methods for viral detection and typing, but third-generation sequencing such as Oxford Nanopore Sequencing, ONT, is becoming a common method for epidemiological studies (e.g., to elucidate viral recombination) [228,229,230,231]. Flint and colleagues used a pre-amplification step to obtain full-length genomic amplicons followed by sequencing on Illumina and ONT platforms in combination to obtain accurate data while reducing the required viral titre [232]. In general, ONT can generate long reads up to 100s of kilobases in a relatively short time and at low cost; however, it is less accurate with a higher error rate compared to Illumina [233,234].

### 3.5. Emerging Molecular Methods

The rapid and early detection of foodborne pathogens is essential for food safety and for outbreak investigations. The most commonly used methods for nucleic acid detection are PCR-based methods thanks to their accuracy. An important limitation of PCR is the expensive instrumentation and special expertise/trained personnel. Simpler approaches allow more rapid, ease of use methods with less cost (on-site testing). Several techniques have been established over time for nucleic acid-based diagnostics. The main types of these methods are recombinase polymerase amplification (RPA), isothermal-based amplification methods (e.g., loop-mediated isothermal amplification, LAMP), and CRISPR-Cas-based detection methods [235]. Reverse transcription loop-mediated isothermal amplification (RT-LAMP) combines LAMP DNA detection with reverse transcription and can be an alternative to RT-qPCR. In the case of LAMP, the nucleic acid amplification is conducted at a constant temperature (between 60 and 65 °C), so a thermal cycler is not needed. Wu and colleges have developed an RT-LAMP assay for the detection of HAV in different food matrices, including green onion, strawberry, mussel, and milk [236]. Their assay showed comparable sensitivity with RT-qPCR in certain food matrices. The specific amplification of the LAMP method relies on four or six designed primers that bind to six regions specific to the target gene [235]. The mismatches between primers and templates significantly reduce the amplification efficiency of LAMP (especially the two inner LAMP primers FIP and BIP that are typically over 40 nt long, which form mismatches easily with templates of highly variable viruses) [237]. The LAMP assay performance on samples varies according to the level of detectable RNA, and the diagnostic performance may change over time from symptom onset in real-life clinical settings [238]. Generally, this technique has great potential, but further improvement and implementation are needed until more extended application. 

Clustered Regularly Interspaced Short Palindromic Repeats (CRISPR)-associated nuclease (Cas) (CRISPR-Cas)-based methods are a promising alternative for pathogen diagnostics. Approaches such as SHERLOCK (based on CRISPR-Cas13a) and DETECTR (based on CRISPR-Cas12a) are potential methods for the rapid detection and identification of infectious diseases, with simple yet ultra-sensitive tests [239,240,241]. Although these methods are rapidly evolving, there are still many challenges that need to be solved before CRISPR sensing can replace or complement established techniques such as PCR. These challenges mainly are sequence limitations, standardisation, quantification and multiplexing.

## 4. Concluding Remarks

The globalisation of the food industry favours the foodborne-related HAV outbreaks, as many high-risk products (shellfish, fresh or frozen fruits, and vegetables) are produced in HAV-endemic countries and imported to countries of low endemicity (e.g., Europe). A standardised method helps the accurate quantification and comparison of analysis results between laboratories and between different survey studies. The available ISO method for the detection of HAV in food gives an opportunity for more harmonised and reliable food control; however, even when harmonised standards are used, considerable variation may occur in results [242]. 

The reported HEV cases in Europe have increased tenfold in the last 20 years partially due to the increasing awareness and the improvement of the detection methods [3]. Yet, the global burden of the disease is considered underestimated and largely unknown. It is difficult to estimate the overall prevalence of HEV, since the methods used in the survey studies are different as well as the samples sizes of these studies. The prevalence varies widely between geographical regions and study populations [243]. A standardised method for HEV detection followed by further research and survey studies of HEV would help achieve a better risk assessment of the food and animal products in the transmission of HEV to humans. Comprehensive and comparable surveillance studies in Europe would help obtain a complete picture regarding the presence and global burden of the virus. 

The nucleic acid-based methods (including the most widely used PCR methods) are not able to discriminate between the infectious and the non-infectious viral particles, which may make the interpretation of a positive test result in foods arguable. However, with the usage of proper controls, etc.; the presence of viral RNA suggests that viral contamination of the tested food has occurred somewhere along the supply chain. Viability PCR methods—which amplify nucleic acids only from intact virions—could be an alternative to this problem [244,245], but these methods are not used in routine surveillance currently. 

Further method improvements can lead to two different directions that complement each other. One direction could be the improvement of a more accurate but simple and quick detection of viruses, such as CRIPSR-Cas based methods. These methods are extremely useful when a rapid detection is needed (e.g., crisis situations). The other direction—not replacing but complementing the rapid tests—could be the improvement of NGS methods (e.g., more user-friendly bioinformatics software) to help the outbreak investigations and the characterisation of the virus strains circulating in the human population and in the environment.

## Figures and Tables

**Table 2:** HEV RNA detection in meat products.

Year of Sampling	Type of Samples	Region/Country	No. of Samples	Percentage of HEV RNA Detection	Reference
2013–2014	pork meat cut, ground pork meat, pig liver, pig intestines, raw pork balls, pork sausages, fermented pork products	Nakhon Pathom Province, Thailand	214	0.5% of pork products, 2.0% of pig liver	[125]
2017–2018	marketed meat cuts (beef, chicken, pork)	Uruguaiana, Rio Grande do Sul, Brazil	131	0%	[126]
2022	pork liver pâtés, raw dried hams and raw dried sausages	Belgium	54	65% of the pork liver pâtés, 15% of raw dried hams and 0% of raw dried sausages	[127]
2018	ground pork, liver	Northern California, United States	209	12.6% of ground pork, 45% of pork liver	[128]
2017–2019	sausages	the Netherlands	316		[129]
2019–2020	liver, liver sausage, liver pate	Germany	131	10%, pork livers (5%), liver sausages (13%) and liver pâté samples (15%)	[130]
2017	pork cuts and organs	Spain	450		[131]
2016	liver, liver sausage, liver pate, meat cuts, sausages, wild boar meat		521	12.7% of livers, 70.7% of liverwurst, 68.9% of liver pate, 0% of the pork chops, 0% of fresh sausages, 0% of wild boar meat	[132]
2017–2018	raw bacon, meat cuts, pork and wild boar sausages, salami, wild boar salami	Southern Italy	162	6.3% of wild boar salamis, 0% all other samples	[133]
2017–2018	meat cuts, liver, kidney, blood curd	Hebei Province, China	413	pig liver 6.1%, kidney 3.1%, and blood curd 1.2%	[134]
not detailed	pork meat cuts, livers, intestines, spleens, ureters	China	107	33.3% of meat cuts, 8.3% of pig liver, 18.7% of intestine, 33.3% of spleen, 26.3% of ureter	[135]
2016	liver sausage, raw meat sausage	Switzerland	90	18.9% of liver sausages, 5.7% of raw meat sausages	[136]
2014–2015	liver sausage, salami, wild boar sausages	Germany	120	20% of raw sausages, 22% of liver sausages	[137]
2011	sausage, liver sausage, dry salted liver, liver quenelles	France	394	30% of sausage, 29% of liver sausages, 25% of liver quenelles, 3% of dried salted liver	[138]

## Data Availability

Not applicable.
